# Clinicopathological characteristics and prognosis of triple-negative breast cancer invasive ductal carcinoma with ductal carcinoma in situ

**DOI:** 10.1007/s00432-023-04895-9

**Published:** 2023-06-24

**Authors:** Yang Liu, Tong Yu

**Affiliations:** Department of Breast Surgery, Harbin Medical University Cancer Hospital, 150 Haping Road, Harbin, 150040 China

**Keywords:** TNBC, IDC-DCIS, Clinicopathological characteristics, Prognosis

## Abstract

**Purpose:**

The purpose of this study is to compare and analyze the clinicopathological characteristics and prognosis of patients with invasive ductal carcinoma coexisting with ductal carcinoma in situ (IDC-DCIS) and invasive ductal carcinoma (IDC) in triple-negative breast cancer (TNBC), and to explore the factors affecting the prognosis, so as to provide new ideas for clinical diagnosis and treatment of these patients.

**Methods:**

The patients with TNBC underwent surgery in the Department of Breast Surgery of Harbin Medical University Cancer Hospital from October 2012 to December 2018 were retrospectively analyzed and divided into IDC-DCIS group and IDC group. The clinicopathological characteristics and prognosis of the two groups were compared. P < 0.05 was considered statistically significant.

**Results:**

A total of 358 patients were enrolled. There were significant differences in age (P = 0.002), family history (P = 0.016), menopausal status (P = 0.003), KI-67% (P < 0.001), lymphovascular invasion (P = 0.010), histologic grade of IDC (P < 0.001) and multifocal (P < 0.001) between the two groups. The disease-free survival (DFS) of the IDC-DCIS group was better than that of the IDC group (the 5-year DFS was 87.9% vs. 82.6%, P = 0.045), but the overall survival (OS) of the two groups was not statistically significant (the 5-year OS was 96.2% vs. 96.0%, P = 0.573). In addition, the coexistence of DCIS (P = 0.030), lymph node pathologic stage (P = 0.001), tumor location (P = 0.011), and adjuvant chemotherapy (P < 0.001) were independent prognostic factors for DFS.

**Conclusion:**

In TNBC, the IDC-DCIS group had less invasive biological characteristics. The DFS of the IDC-DCIS group was better than that of the IDC group, but there was no statistical difference in OS between the two groups. In addition, the coexistence of DCIS, lymph node stage, tumor location and adjuvant chemotherapy may be independent prognostic factors for DFS.

## Introduction

Breast cancer has a high incidence among female cancer patients in the world, and its related mortality has jumped to the second place (Siegel et al. 2021; Bray et al. 2018). According to the expression of ER, PR, Her-2 and KI-67, breast cancer has been classified into four types: luminal A type, luminal B type, Her-2 overexpression type and triple negative type (Lam et al. 2014). TNBC is a special molecular subtype, meaning that ER, PR and Her-2 are all expressed negatively, and accounts for 15% of all breast cancers. Its high invasiveness and susceptibility to recurrence and metastasis result in the worst prognosis of all subtypes (Tariq and Rana 2013; Foulkes et al. 2010; Stagg and Allard 2013).

DCIS refers to the proliferation of tumor epithelial cells in the ductal lobular system. These abnormal epithelial cells have the morphological characteristics of invasive carcinoma of the breast, but they are different from invasive carcinoma. It is surrounded by myoepithelial cells and basement membrane of the duct and does not invade the stroma or lymphatic vessels or blood vessels (Pang et al. 2016). However, DCIS itself does not cause metastatic disease or death, it is regarded as a non-specific precursor of invasive breast cancer (Lagios and Silverstein 2015). IDC is the most common type of breast cancer, which is formed by DCIS breaking through the basement membrane of the ductule, lobular terminal duct or acini to the surrounding stroma. A number of studies have confirmed that some DCIS will eventually develop into IDC, and in this infiltration process, about 45% of IDC may coexist with DCIS, and its clinicopathological characteristics and prognosis are different from those of pure IDC (Wong et al. 2010; Ruszczyk et al. 2016). Some studies have found that the IDC-DCIS group has weaker invasive biological characteristics than the IDC group. Compared with IDC patients, the prognosis of IDC-DCIS patients was significantly improved (Lopez Gordo et al. 2019; Goh et al. 2019; Kole et al. 2019; Chen et al. 2019a; Wong et al. 2012; Guan et al. 2020; Carabias-Meseguer et al. 2013). Triple-negative IDC is less commonly coexisted with DCIS. Therefore, the clinicopathological characteristics and prognosis of this type of patients have not been studied. In addition, there is no clear treatment plan for this type of patients, only treating IDC-DCIS as IDC, so we don't know whether it caused overtreatment for this type of patients. In order to achieve "individualized" and "accurate" treatment, we should skillfully master the characteristics and prognosis of all types of breast cancer. Therefore, this paper studies the clinicopathological characteristics and prognosis of triple negative breast cancer IDC-DCIS. The purpose of this study was to compare and analyze the clinicopathological characteristics and prognosis of triple-negative invasive ductal carcinoma coexisting with ductal carcinoma in situ (IDC-DCIS) and pure invasive ductal carcinoma (IDC), and to explore the factors affecting the prognosis, in order to provide new ideas for the clinical diagnosis and treatment of these patients.

## Methods

### Patient population

The clinicopathological data of patients with TNBC in the Department of Breast Surgery of our hospital from October 2012 to December 2018 were selected and divided into IDC-DCIS group and IDC group according to their histological types.

The inclusion criteria were as follows: the patients with primary breast cancer were pathologically diagnosed after surgery, and the immunohistochemistry was triple negative; the histological type was pure IDC or IDC-DCIS; the tumor pathological stage was pT1-T3, and the lymph node pathological stage was pN0-pN3; unilateral breast cancer without distant metastasis before treatment; the clinicopathological data and follow-up information were complete.

Exclusion criteria were as follows: those who had received neoadjuvant chemotherapy (NAC); non-primary breast cancer or combined with other malignancies; immunohistochemical type or Her-2 positive; histologic types except simple IDC and IDC-DCIS; pT4; distant metastasis before treatment; bilateral breast cancer; clinicopathological data and follow-up information was incomplete.

### Pathological evaluation criteria

The pathological sections were independently reviewed by experienced pathologists. The results of ER and PR showed that the proportion of positive cells ≥ 1% was positive, and the proportion of positive cells less than 1% was negative. Evaluation of the Her-2 result: According to the guidelines for Her-2 detection in breast cancer, it was negative 0; negative 1; uncertain 2; positive 3 + . For the areas with uncertain 2 + , the final results were positive or negative by FISH (in situ hybridization). The results of KI-67 showed that the positive rate of tumor nuclei cells ≥ 20% was high expression, and that of tumor nuclei cells < 20% was low expression. According to the Nottingham grading system, the histological grading system of IDC can be divided into low (G1 level), intermediate (G2 level) and high (G3 level). According to the criteria established by AJCC, the TNM pathological staging of breast cancer has been adopted. In addition, for cases of invasive ductal carcinoma with ductal carcinoma in situ, the pathological results are based on the invasive components.

### Statistical analysis

The data of this study were statistically analyzed using SPSS 26.0 software. T-test is used to compare the differences between the two groups for the measurement data that conform to the normal distribution; Mann‐Whitney U test is used to compare the differences between the two groups for the measurement data that do not conform to the normal distribution; Chi-square test is used to compare the count data of the two groups. The survival curve was plotted using GraphPadPrism9.0 software and analyzed using the Kaplan–Meier method, and the difference in survival between the two groups was analyzed using the log-rank test. The primary endpoint of this study was DFS, defined as the time from diagnosis of breast cancer to first breast cancer recurrence, distant metastasis, or no breast cancer recurrence to death. The secondary endpoint was OS, defined as the time from diagnosis of breast cancer to death from any cause. Univariate and multivariate Cox regression were used to determine the hazard ratio (HR), 95% confidence interval (CI), and risk factors associated with survival. P < 0.05 was considered statistically significant.

## Results

### Patients’ clinicopathological characteristics and distribution

From October 2012 to December 2018, a total of 358 patients met the inclusion criteria and had complete data. According to the postoperative pathological report, the patients were divided into IDC-DCIS group and IDC group. Among them, there were 169 cases in IDC-DCIS group and 189 cases in IDC group. The mean age of the IDC-DCIS group was 53 years old, and that of the IDC group was 49 years old (P = 0.002). There are 11 cases (6.5%) in the IDC-DCIS group had family history, 3 cases (1.6%) in the IDC group had family history (P = 0.016); In the IDC-DCIS group, 117 cases (69.2%) had a KI-67% of 20% or higher, while in the IDC group, 178 cases (94.2%) had a KI-67% of 20% or higher (P < 0.001). In the IDC-DCIS group, 18 cases (10.7%) showed lymphovascular invasion, while in the IDC group, 39 cases (20.6%) showed the same (P = 0.010); in the IDC-DCIS group, 4 cases (2.4%) were classified as histologic grade G1, 117 cases (69.2%) as histologic grade G2, and 48 cases (28.4%) as histologic grade G3. In comparison, in the IDC group, 33 cases (17.5%) were classified as histologic grade G2 and 156 cases (82.5%) were classified as histologic grade G3 (P < 0.001); in the IDC-DCIS group, 17 cases (10.1%) were identified as multifocal, whereas in the IDC group, only 2 cases (1.1%) were identified as multifocal (P < 0.001). (The clinicopathological characteristics and distribution of the two groups are detailed in Table 1).

**Table 1:** Clinicopathological characteristics of the TNBC IDC-DCIS and IDC groups

Variable	IDC-DCIS (n = 169) (%)	IDC (n = 189) (%)	*P* value
Age (years), mean ± SD	53 ± 10.73	49 ± 9.30	0.002
Lactation history			0.159
Yes No	148 (87.6) 21 (12.4)	174 (92.1) 15 (7.9)	
Family history			0.016
Yes No	11 (6.5) 158 (93.5)	3 (1.6) 186 (98.4)	
Menopausal status			0.003
Pre Post	62 (36.7) 107 (63.3)	99 (52.4) 90 (47.6)	
BMI (kg/m2), median	23.0	23.0	0.32
pT stage			0.511
pT1 pT2 pT3	97 (57.4) 69 (40.8) 3 (1.8)	103 (54.5) 79 (41.8) 7 (3.7)	
pN stage			0.147
pN0 pN1 pN2 pN3	120 (71.0) 34 (20.1) 12 (7.1) 3 (1.8)	132 (69.8) 36 (19.0) 9 (4.8) 12 (6.3)	
KI-67% index			< 0.001
< 20% ≥ 20%	52 (30.8) 117 (69.2)	11 (5.8) 178 (94.2)	
Lymphovascular invasion			0.010
Yes No	18 (10.7) 151 (89.3)	39 (20.6) 150 (79.4)	
Histological grade			< 0.001
Low grade (G1)	4 (2.4)	None	
Median grade (G2)	117 (69.2)	33 (17.5)	
High grade (G3)	48 (28.4)	156 (82.5)	
Tumor location			0.395
Upper-outer quadrant Upper-inner quadrant Lower-outer quadrant Lower-inner quadrant Central portion	114 (67.5) 26 (15.4) 19 (11.2) 3 (1.8) 7 (4.1)	125 (66.1) 27 (14.3) 18 (9.5) 11 (5.8) 8 (4.2)	
Tumor focality			< 0.001
Unifocal Multifocal	152 (89.9) 17 (10.1)	187 (98.9) 2 (1.1)	
Breast surgery			0.248
Mastectomy BCS Breast reconstruction	153 (90.5) 16 (9.5) None	170 (89.9) 16 (8.5) 3 (1.6)	
ALND			0.781
Yes No	95 (56.2) 74 (43.8)	109 (57.7) 80 (42.3)	
Chemotherapy			0.582
Yes No	165 (97.6) 4 (2.4)	187 (98.9) 2 (1.1)	
Radiotherapy			0.992
Yes No	43 (25.4) 126 (74.6)	48 (25.4) 141 (74.6)	

## Survival outcomes

Follow-up has ended up in October 2022, with a median of 69 months, a minimum of 10 months and a maximum of 122 months. A total of 58 patients experienced endpoint events related to recurrence, distant metastasis or death. Of these, 26 patients experienced recurrence, 32 patients experienced distant metastasis and 14 patients died. Among the patients who experienced metastasis, 4 had liver metastasis, 8 had lung metastasis, 13 had bone metastasis, 3 had brain metastasis and 4 had multiple organ metastasis. In the IDC-DCIS group, 9 recurrences, 10 metastases (including 3 bone metastases, 1 brain metastases, 2 liver metastases, 2 lung metastases, and 2 multiple organ metastases), and 5 deaths were observed. In the IDC group, 17 recurrences, 22 metastases (including 10 bone metastases, 2 brain metastases, 2 liver metastases, 6 lung metastases, and 2 multiple organ metastases), and 9 deaths were observed. (The recurrence and survival outcomes are detailed in Table 2).

**Table 2:** Recurrence and survival outcomes between patients with IDC-DCIS and IDC

	IDC-DCIS (%)	IDC (%)	Total (%)
Local/regional/contralateral recurrence	9 (5.7)	17 (9.0)	26 (7.0)
Metastasis Bone Brain Liver Lung Mixed Death	10 (5.9) 3 (1.7) 1 (0.5) 2 (1.1) 2 (1.1) 2 (1.1) 5 (2.9)	22 (11.6) 10 (5.3) 2 (1.1) 2 (1.1) 6 (3.2) 2 (1.1) 9 (4.8)	32 (8.9) 13 (3.6) 3 (0.8) 4 (1.1) 8 (2.2) 4 (1.1) 14 (3.9)

**Fig. 1:**
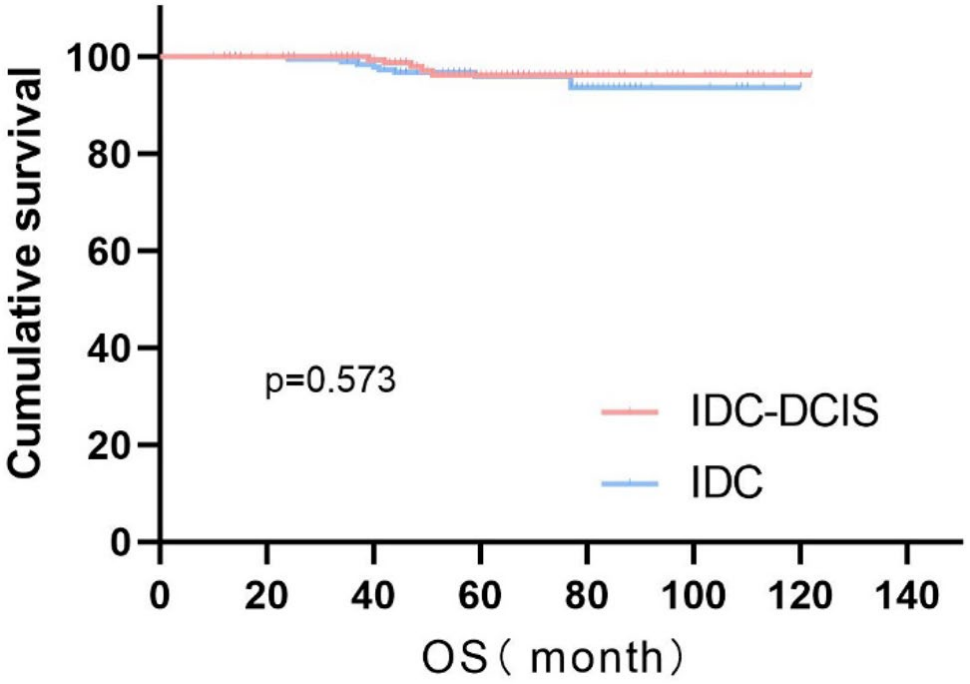
Kaplan–Meier Curves of OS for IDC-DCIS and IDC groups in TNBC

**Fig. 2:**
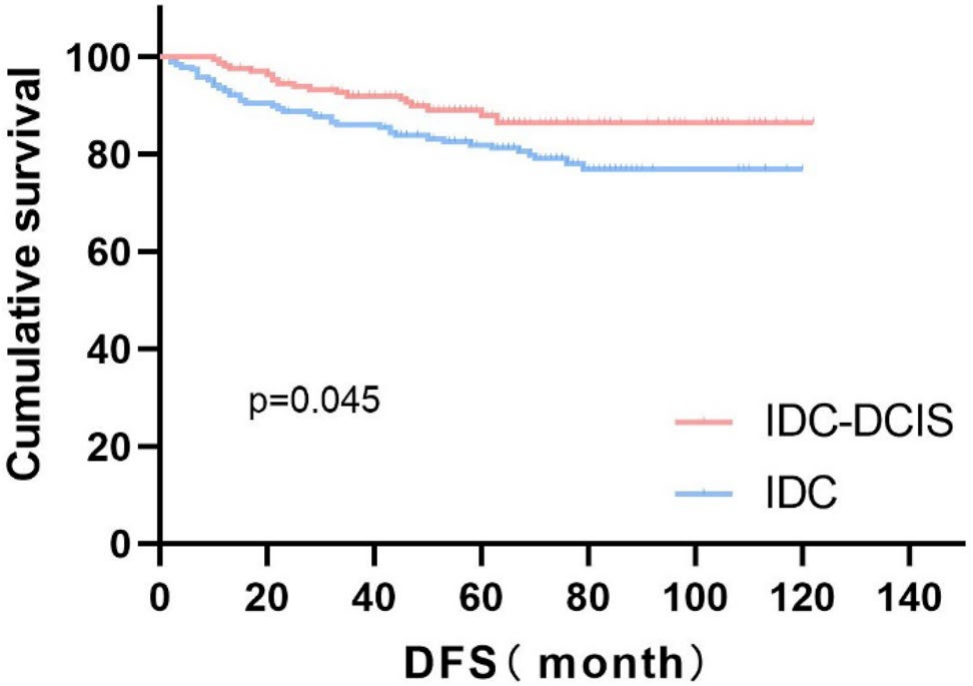
Kaplan–Meier Curves of DFS for IDC-DCIS and IDC groups in TNBC

In this study, the Kaplan–Meier method was used to analyze survival time. The results showed that there was no significant difference in OS between the IDC-DCIS group and the IDC group, with both groups having a 5-year OS of 96.2% vs. 96.0%, P = 0.573 (Fig. 1). However, a statistically significant difference in DFS was observed between the two groups, with the IDC-DCIS group having a better 5-year DFS of 87.9% compared to 82.6% in the IDC group, P = 0.045 (Fig. 2). These results suggest that the IDC-DCIS group had better disease-free survival than the IDC group.

**Fig. 3:**
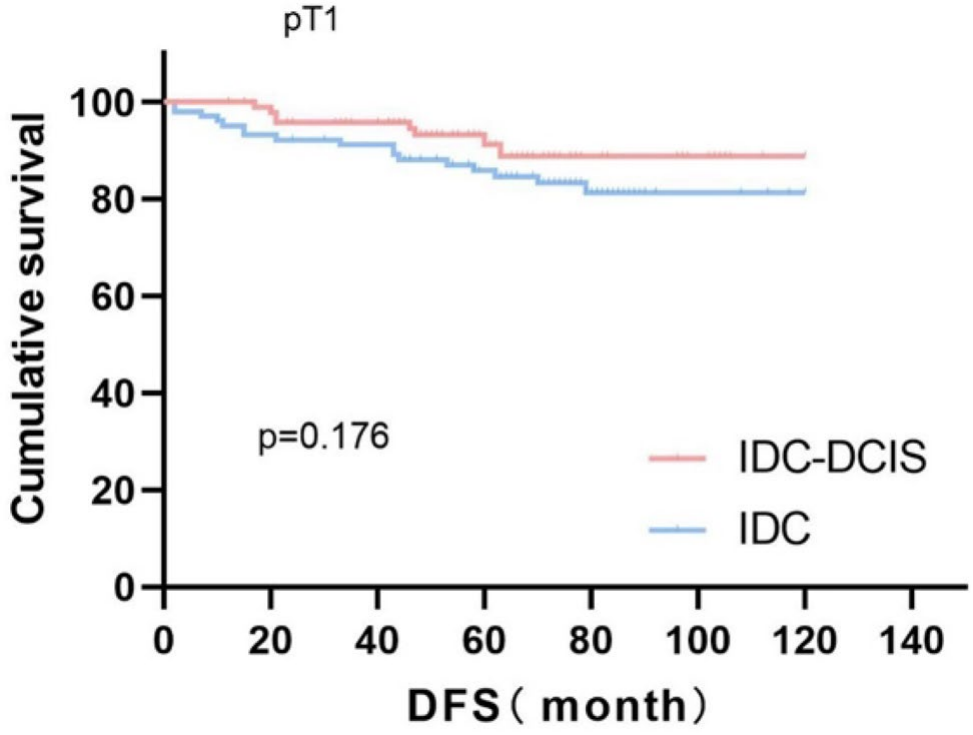
Kaplan–Meier Curves of DFS for IDC-DCIS and IDC groups in TNBC

**Fig. 4:**
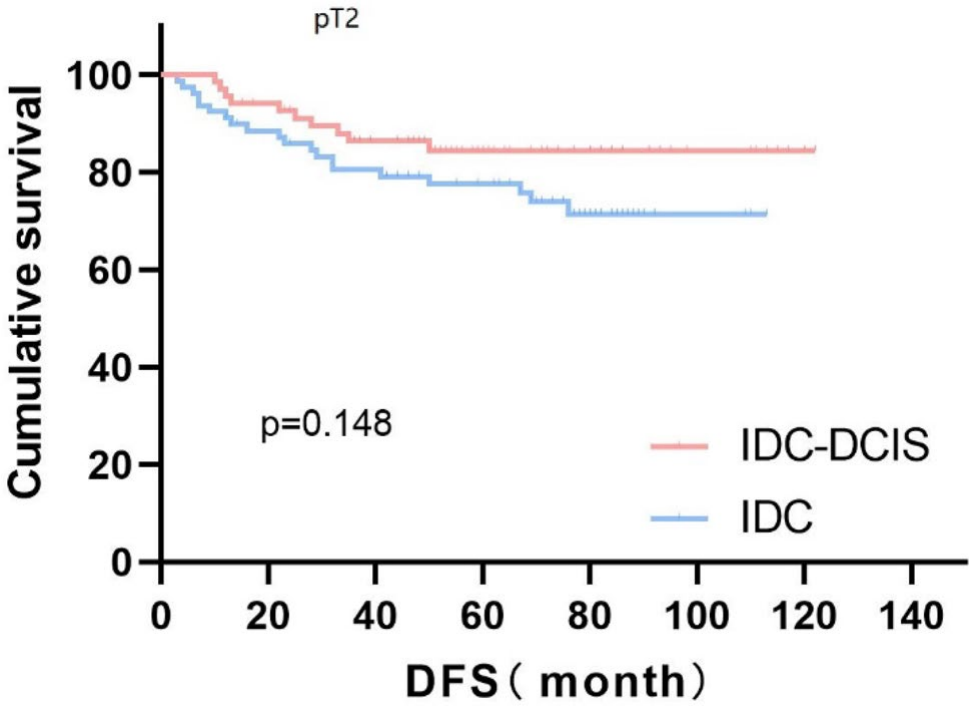
Kaplan–Meier Curves of DFS for IDC-DCIS and IDC groups in TNBC

**Fig. 5:**
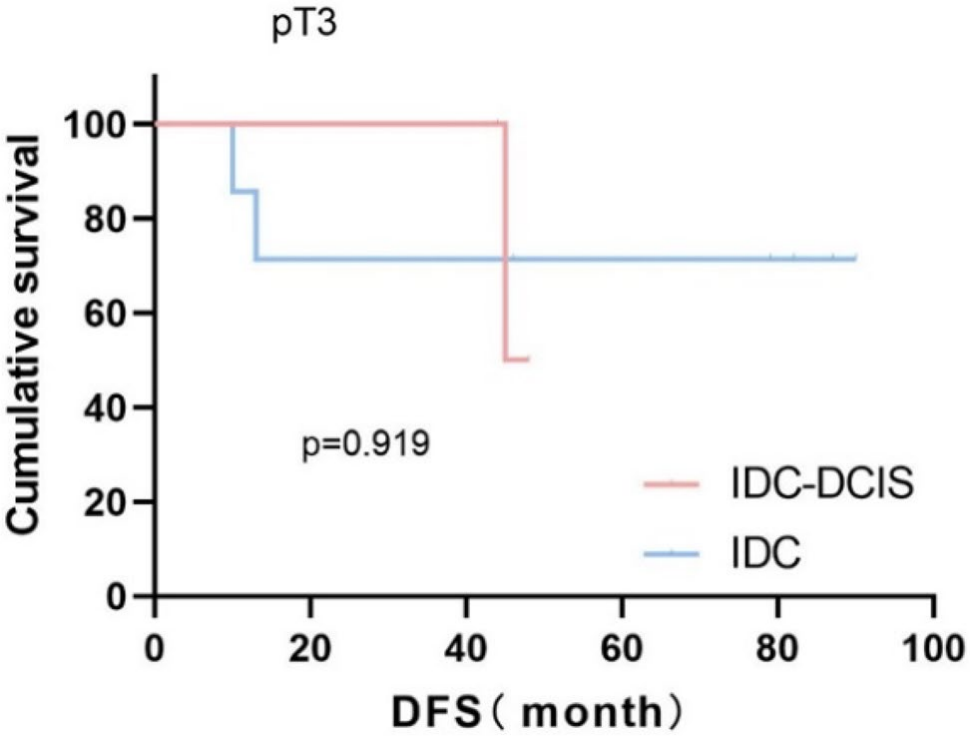
Kaplan–Meier Curves of DFS for IDC-DCIS and IDC groups in TNBC

**Fig. 6:**
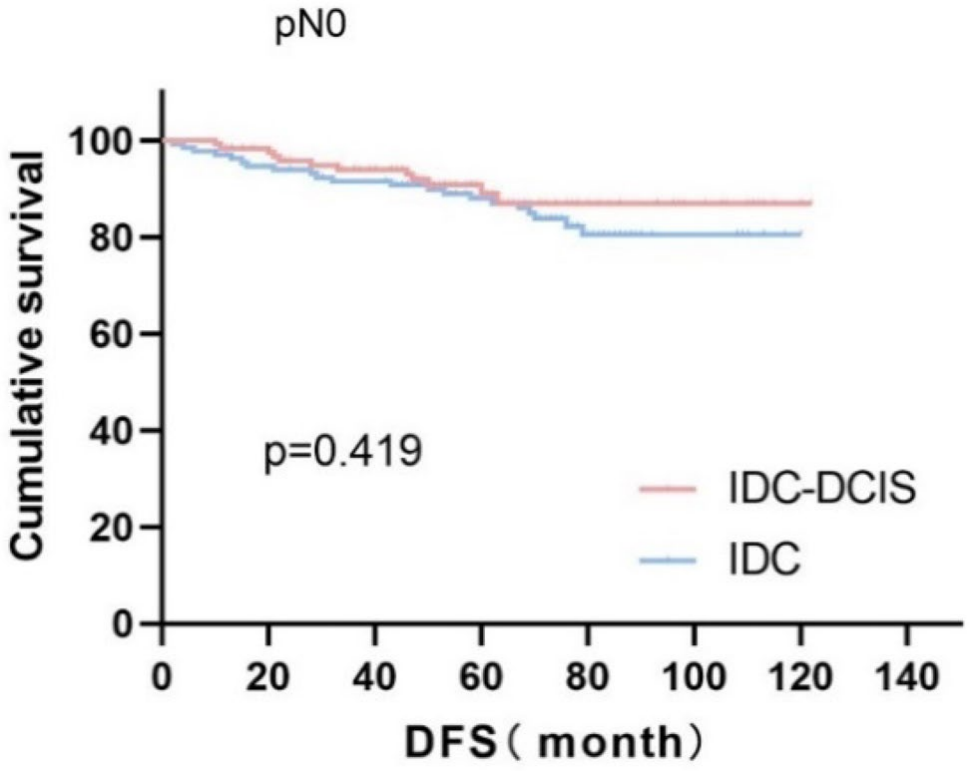
Kaplan–Meier Curves of DFS for IDC-DCIS and IDC groups in TNBC

**Fig. 7:**
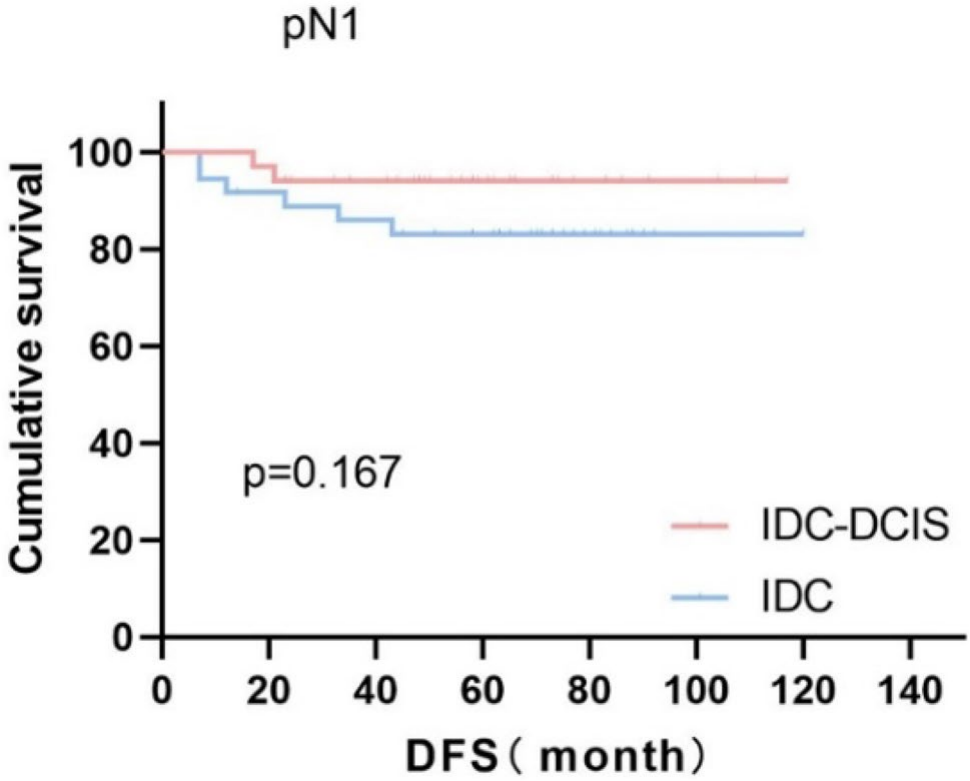
Kaplan–Meier Curves of DFS for IDC-DCIS and IDC groups in TNBC

**Fig. 8:**
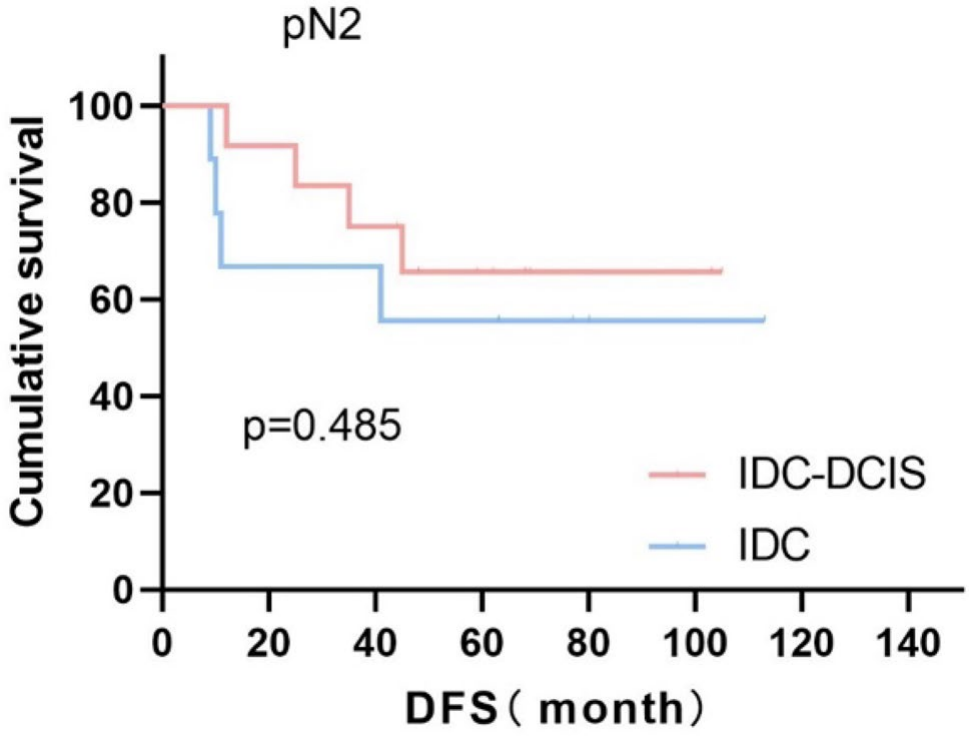
Kaplan–Meier Curves of DFS for IDC-DCIS and IDC groups in TNBC

**Fig. 9:**
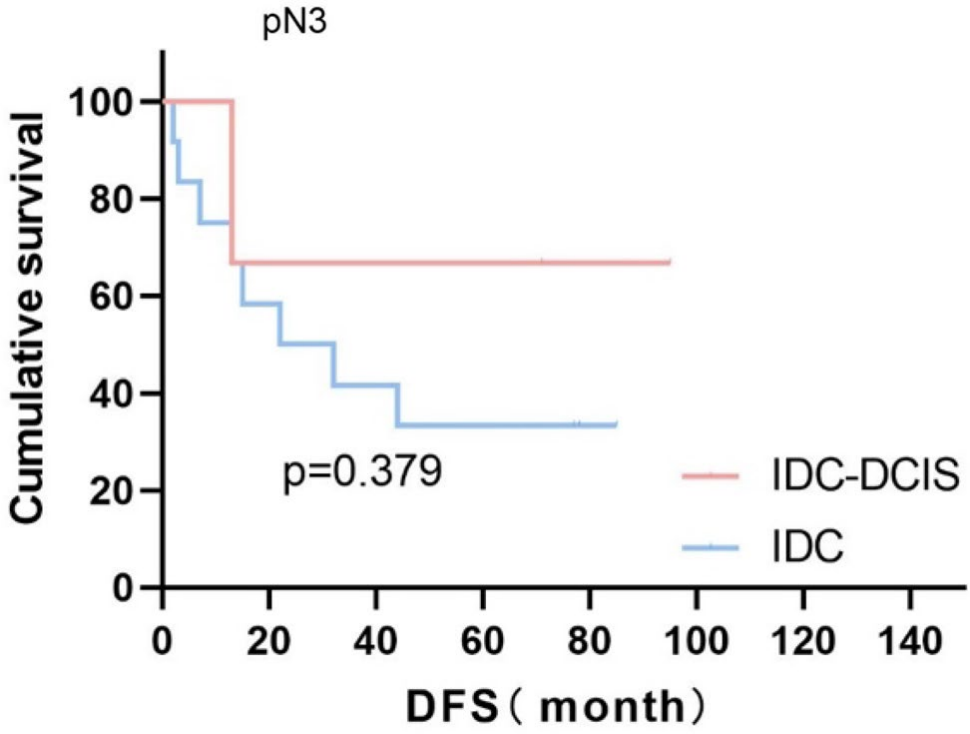
Kaplan–Meier Curves of DFS for IDC-DCIS and IDC groups in TNBC

**Fig. 10:**
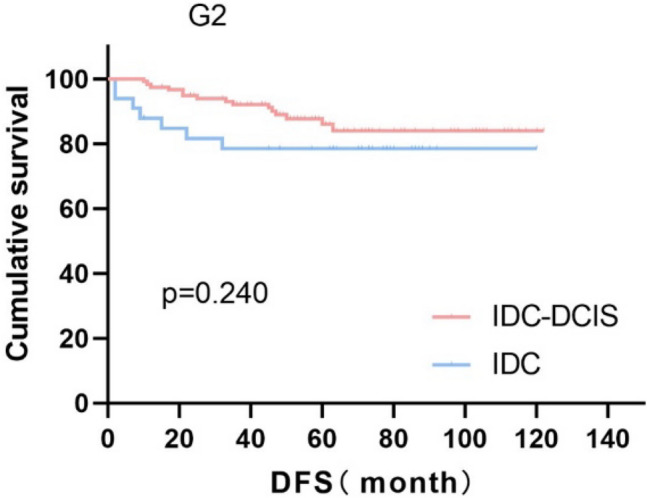
Kaplan–Meier Curves of DFS for IDC-DCIS and IDC groups in TNBC

**Fig. 11:**
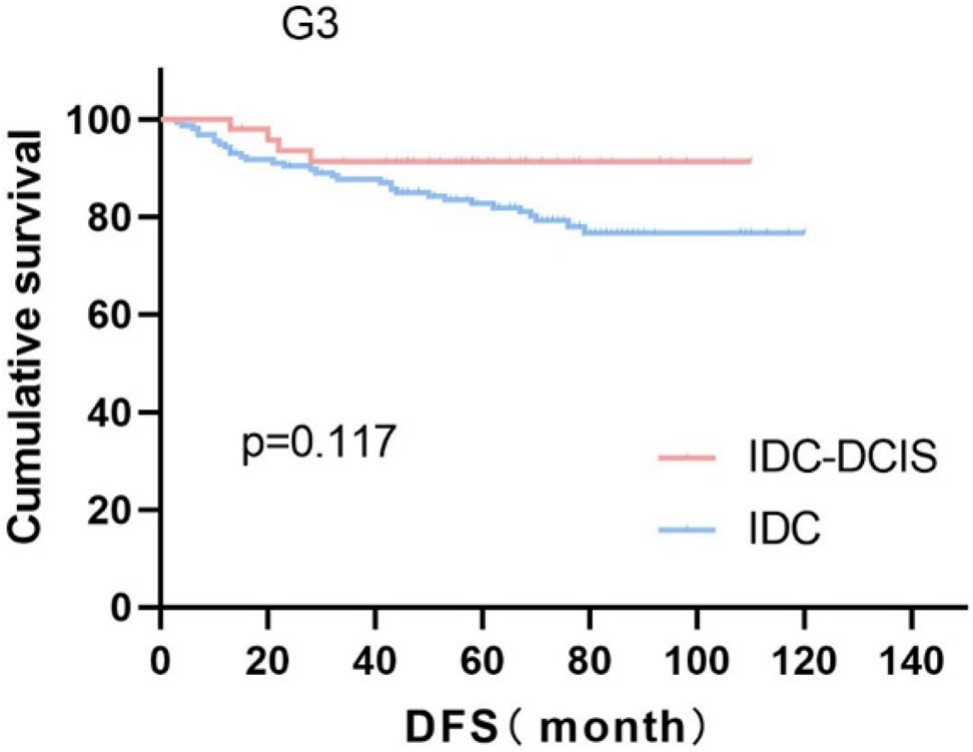
Kaplan–Meier Curves of DFS for IDC-DCIS and IDC groups in TNBC

Survival curves were plotted using pT stage, pN stage and histologic grade as stratification factors, and survival time was analyzed by the Kaplan–Meier method. The 5-year DFS in IDC-DCIS group vs. IDC group was 91.1% vs. 85.8% for pT1, p = 0.176 (Fig. 3); 84.4% vs. 77.7% for pT2, p = 0.148 (Fig. 4); and 50% vs. 71.4% for pT3, p = 0.919 (Fig. 5). The 5-year DFS in both groups was 88.9% vs. 87.9% for pN0, P = 0.419 (Fig. 6); 94.1% vs. 83.1% for pN1, P = 0.167 (Fig. 7); 65.6% vs. 55.6% for pN2, P = 0.485 (Fig. 8); for pN3, 66.7% vs. 33.3%, P = 0.379 (Fig. 9). Since histologic grade G1 was not present in the IDC group, histologic grade G2 and G3 were used as stratification factors for the comparative analysis of survival time between the two groups. As shown in Fig. 10, the 5-year DFS in both groups was 85.9% vs. 78.5% for grade G2, P = 0.240; by Fig. 11, it was found that the 5-year DFS in both groups was 91.3% vs. 82.6% for grade G3, P = 0.117. None of the above P values were statistically significant, which may be related to the small sample size of the stratified study.

### Cox univariate and multivariate analysis affecting DFS.

Table 3 shows the results of both univariate and multivariate analyses of DFS risk factors. Univariate analysis identified several risk factors affecting DFS, including coexistence of DCIS (P = 0.048), pathological lymph node stage (P < 0.001), lymphovascular invasion (P = 0.019), tumor location (P = 0.011), adjuvant chemotherapy (P = 0.002), and radiotherapy (P = 0.011). On the other hand, multivariate analysis revealed that the coexistence of DCIS (P = 0.030), pathological lymph node stage (P = 0.001), tumor location (P = 0.011), and adjuvant chemotherapy (P < 0.001) were independent prognostic factors significantly affecting DFS.

**Table 3:** Cox univariate and multivariate regression analysis of risk factors for DFS

	Univariate analysis			Multivariate analysis		
	HR	*P* value	95%CI	HR	*P* value	95%CI
Age	1.023	0.091	0.996–1.050			
Lactation history						
No vs Yes	0.475	0.210	0.149–1.520			
Family history						
No vs Yes	2.477	0.369	0.343–17.889			
Menopausal status						
Pre vs Post	0.893	0.668	0.532–1.499			
Groups						
IDC-DCIS vs IDC	0.574	0.048	0.331–0.996	0.535	0.030	0.304–0.942
BMI	1.009	0.815	0.939–1.084			
pT stage		0.062				
pT1 vs pT3	0.370	0.103	0.111–1.225			
pT2 vs pT3	0.644	0.468	0.197–2.112			
pN stage		< 0.001			0.001	
pN0 vs pN3	0.051	< 0.001	0.072–0.318	0.159	0.001	0.056–0.448
pN1 vs pN3	0.136	< 0.001	0.052–0.354	0.165	0.001	0.059–0.460
pN2 vs pN3	0.530	0.193	0.204–1.377	0.556	0.248	0.206–1.505
KI-67% index						
< 20% vs ≥ 20%	1.009	0.979	0.511–1.996			
Lymphovascular invasion						
No vs Yes	0.496	0.019	0.275–0.893	0.875	0.711	0.431–1.774
Histological grade						
Low vs High	< 0.001	0.971	0.000–2.816E + 251			
Median vs High	0.877	0.629	0.516–1.492			
Tumor location					0.011	
Upper-inner vs Upper-outer	2.028	0.027	1.085–3.790	2.364	0.010	1.227–4.554
Lower-outer vs Upper-outer	0.747	0.582	0.265–2.109	0.784	0.650	0.274–2.244
Lower-inner vs Upper-outer	0.470	0.457	0.064–3.436	0.259	0.194	0.034–1.987
Central portion vs Upper-outer	3.494	0.005	1.461–8.355	2.654	0.034	1.076–6.545
Tumor focality						
Unifocal vs Multifocal	2.826	0.304	0.391–20.440			
Breast surgery						
Mastectomy vs Breast reconstruction	0.359	0.433	0.049–2.604			
BCS vs Breast reconstruction	0.506	0.311 0.525	0.062–4.125			
ALND						
Yes vs No	1.385	0.239	0.085–2.381			
Chemotherapy						
No vs Yes	6.195	0.002	1.922–19.969	10.134	< 0.001	2.953–34.780
Radiotherapy						
No vs Yes	0.505	0.011	0.298–0.854	0.856	0.674	0.415–1.765

## Discussion

DCIS is considered a non-specific precursor of IDC (Lagios and Silverstein 2015), and approximately 30% of DCIS may progress to IDC (Kroman et al. 2003). Studies have found that in IDC-DCIS, the proportion of triple-negative molecular types is relatively small, while the proportion of Her-2 positive and HR positive patients is relatively large (Goh et al. 2019; Kole et al. 2019; Guan et al. 2020; Carabias-Meseguer et al. 2013). In addition, among TNBCs, 97.9% of DCIS can progress to IDC, of which 62.6% progress to IDC (Thike et al. 2013). The pathogenesis of breast cancer is still unclear, and two mechanisms are generally considered: one is independent lineage, in which DCIS and IDC tumors in the same individual normal breast tissue proliferate from two different progenitor cells. This mechanism assumes that DCIS and IDC evolved independently and that the cell lines do not share overlapping mutations or gene copy number variants. Miron et al. sequenced PIK3CA mutations in DCIS and IDC matched patients and found only 30% concordance between in situ and infiltrative region (Miron et al. 2010). Foschini et al. performed deep sequencing of mitochondrial D-loops in DCIS patients and found that 61% of tumors were of non-clonal or independent origin (Foschini et al. 2013). The limited description of other genomes in these studies has led to a slight lack of evidence for this doctrine. The more accepted pathogenesis of breast cancer is the direct spectrum, in which DCIS and IDC tumors proliferate from a single normal cellular origin. The direct spectrum is divided into two types of invasion: evolutionary bottleneck and polyclonal invasion. Evolutionary bottleneck proposes that during invasion, clones are selected in the duct and migrate to adjacent tissues to form invasive tumors, whereas polyclonal invasion proposes that invasion occurs by the escape of multiple clones from the duct in a coordinated process or by random escape following basement membrane degradation (Casasent et al. 2017, 2018). A meta-analysis pooled data from 38 studies and found that 67% of them supported a direct genealogy, which is now generally accepted as DCIS evolving into IDC (Rebbeck et al. 2022). At the epigenetic level, promoter hypermethylation may play a role in DCIS progression (Johnson et al. 2015; DeVaux and Herschkowitz 2018). The tumor microenvironment also plays an important role in the infiltrative transformation of DCIS. During the progression of DCIS to IDC, significant changes occur in various types of tumor microenvironment, including cancer-associated fibroblasts (CAFs), myoepithelial cells (MECs), and immune cells (Bu et al. 2019; Dawoud et al. 2022; Gil Del Alcazar et al. 2020). In addition, it has also been found that immune cell infiltration is also critical in promoting DCIS infiltrative transformation (Gil Del Alcazar et al. 2020; Chen et al. 2019b; Kim et al. 2020; Niwińska and Olszewski 2021). In recent years, there have also been several studies on important markers during DCIS infiltrative transformation (Yu et al. 2019; Elsarraj et al. 2020; Kim et al. 2021). In conclusion, the specific mechanisms of infiltrative transformation of ductal carcinoma in situ remain to be explored. However, the presence of DCIS in IDC has not clearly influenced the prognosis and treatment strategies. This study analyzed the difference in clinicopathological characteristics and prognosis between the IDC-DCIS group and the IDC group.

In our study, the mean age was older in the IDC-DCIS group than in the IDC group (53 vs 49, P = 0.002). The IDC-DCIS group was more likely to have a family history (6.5% vs. 1.6%, P = 0.016). The IDC-DCIS group was more likely to be postmenopausal (63.3% vs. 47.4%, P = 0.003). Similar results were also reported by Goh et al. KI-67% was more highly expressed in the IDC group (94.2% vs 69.2%, P < 0.001). Wong et al. retrospectively analyzed the pathological data of 1355 cases of IDC-DCIS and IDC and obtained similar results (Wong et al. 2012). Lymphovascular invasion was less in the IDC-DCIS group than in the IDC group (10.7% vs. 20.5%, P = 0.011). Similar results were reported by Guan et al. (Guan et al. 2020) The histologic grade of the IDC-DCIS group was lower than that of the IDC group, and the difference was statistically significant (P < 0.001), suggesting that the histologic grade of IDC was higher and more aggressive. In the IDC-DCIS group, 17 patients (10.1%) were multifocal, while only 2 patients (1.1%) in the IDC group were multifocal, and the difference was statistically significant (P < 0.001). The appearance of this result may be related to the multifocal nature of pure DCIS. Goh et al. also had similar results, but they believed that this was related to the fact that more patients with IDC-DCIS chose mastectomy (Goh et al. 2019). In addition, a study analyzed the association between 21-gene recurrence scores and IDC-DCIS. 21-gene recurrence scores were lower in IDC-DCIS, which may be associated with reduced expression of proliferative and invasive genes, especially when the proportion of DCIS in IDC is high and grading is low. 21-gene recurrence scores were significantly lower in IDC to DCIS ratio ≥ 50% than in IDC-DCIS < 50% of patients. In addition, genes in both the proliferative (including KI-67, CCNB1, and MYBL2) and invasive (MMP11 and CTSL2) groups of the 21-gene group were significantly less expressed in IDC-DCIS tumors than in IDC alone (Zeng et al. 2021). The above results all suggest to a certain extent that the IDC-DCIS group has weaker biological invasiveness.

The study was followed up to the cut-off date and a total of 58 endpoint events occurred. Among them, the IDC-DCIS group had more recurrence, metastasis and death events, suggesting that the prognosis of the IDC group was poor. Figure 1. shows that there is no difference in OS between the IDC-DCIS group and the IDC group, and the 5-year OS is 96.2% vs 96.0%, P = 0.573; as shown in Fig. 2, there is a significant difference in DFS between the two groups, 5-year DFS was 87.9% vs 82.6%, P = 0.045. This shows that the disease-free survival of the IDC-DCIS group was significantly improved. In addition, we also observed that the 10-year DFS of the IDC-DCIS group was 86.5% and the 10-year OS was 96.2%; the 10-year DFS of the IDC group was 76.9% and the 10-year OS was 93.6%. In this study, after pT stage, pN stage and histological grade were stratified to discuss the survival difference, it was found that there was no survival difference between the IDC-DCIS group and the IDC group, which may be related to the small sample size of this study. Many studies have found that DFS and OS of IDC-DCIS patients are significantly improved (Lopez Gordo et al. 2019; Goh et al. 2019; Kole et al. 2019; Chen et al. 2019a; Wong et al. 2012; Guan et al. 2020; Carabias-Meseguer et al. 2013). However, the OS in this study was not significantly improved, which may be related to the fact that the enrolled population was early operable breast cancer. In a study that included 3001 patients with all subtypes, the DFS of Her-2 positive subtype in the IDC-DCIS group was better than that in the IDC group, while there was no significant difference in DFS between the two groups in patients with triple negative molecular subtypes, which may be related to the small sample size of triple negative IDC-DCIS patients (Goh et al. 2019).

Through Cox univariate and multivariate analysis, it was found that the coexistence of DCIS (IDC-DCIS vs. IDC, HR = 0.574, 95%CI 0.331–0.996, P = 0.048), lymph node pathologic stage (N0 vs. N3, HR = 0. 151, 95%CI 0.072–0.318, P = 0.001; N1 vs N3, HR = 0.136, 95%CI 0.052–0.354, P = 0.001), lymphovascular invasion (no vs yes, HR = 0.496, 95%CI 0.275–0.893, P = 0. 019), tumor location (inner upper quadrant vs. outer upper quadrant, HR = 2.028, 95%CI 1.085–3.790, P = 0.027; central area vs. outer upper quadrant, HR = 3.494, 95%CI 1.461–8.355, P = 0. 005), adjuvant chemotherapy (no vs yes, HR = 6.195, 95%CI 1.922–19.969, P = 0.002), adjuvant radiotherapy (no vs yes, HR = 0.505, 95%CI 0.298 -0.854, P = 0.011) are all factors that influence DFS in univariate analysis. Inclusion of these factors in the multivariate analysis revealed that IDC (IDC-DCIS vs. IDC, HR = 0.535, 95%CI 0.304–0.942, P = 0.030), lymph node pathological stage N3 (N0 vs. N3, HR = 0.159, 95%CI 0.056–0.448, P = 0.001; N1 vs. N3, HR = 0.165, 95%CI 0.059–0.460, P = 0.001), tumor location in the upper inner quadrant or central area (upper inner quadrant vs. upper outer quadrant, HR = 2.364, 95%CI 1.227–4.554, P = 0.010; central area vs outer upper quadrant, HR = 2.654, 95%CI 1.076–6.545, P = 0.034), not receiving adjuvant chemotherapy (no vs yes, HR = 10.134, 95% CI 2.953–34.780, P < 0.001) are independent risk factors for DFS. Goh et al. also found that coexistence of DCIS and lymph node status were independent prognostic factors affecting DFS (Goh et al. 2019). In addition, this study found that the risk of recurrence and metastasis in patients with tumors located in the inner and upper quadrants was higher than that in the outer and upper quadrants; the risk of recurrence and metastasis in patients with tumors located in the central area was also higher than that in the outer and upper quadrants. This may be related to the fact that the mass in the upper inner quadrant and the central area is not easy to find. However, the relationship between tumor location and prognosis remains controversial (Kroman et al. 2003; Sarp et al. 2007; Jayasinghe and Boyages 2009). One study analyzed the relationship between tumor location and prognosis in 17,659 patients and ultimately found that patients with positive axillary lymph nodes had a worse prognosis than those with tumors located in the lower outer quadrant and the inner quadrant (Kroman et al. 2003). Sarp et al. found that a mass located in the lower inner quadrant was an independent prognostic factor for patients with stage T1N0M0 (Sarp et al. 2007). However, Jayasinghe et al. believed that the prognosis of patients with tumors located in the medial quadrant was not different from that of patients with tumors located in the lateral quadrant (Jayasinghe and Boyages 2009). Therefore, the effect of tumor location on prognosis needs to be continuously investigated. In addition, because TNBC is characterized by high aggressiveness and poor prognosis, postoperative adjuvant chemotherapy is required. The results of this study showed that not receiving postoperative adjuvant chemotherapy was a risk factor for DFS, suggesting that a small number of patients in this study had poor compliance. To improve patient outcomes, patient compliance also needs to be improved. In conclusion, we need to continue to explore the independent prognostic factors that influence DFS.

This study is a retrospective study and it is a single center, which has limitations, and selection bias will inevitably occur. Secondly, due to the long follow-up period, which leads to a small increase in the loss to follow-up rate, and the small sample size may cause statistical differences, large-scale multicenter prospective randomized controlled trials are still needed for research.

## Conclusion

In TNBC patients, the IDC-DCIS group had less aggressive biological characteristics than the IDC group. The DFS of the IDC-DCIS group was better than that of the IDC group in TNBC, but there was no statistical difference in OS between the two groups. Independent prognostic factors affecting DFS included coexistence of DCIS, lymph node pathologic stage, tumor location, and adjuvant chemotherapy.

## Data Availability

The datasets generated during and/or analyzed during the current study are available from the corresponding author on reasonable request.
